# Assessing the youth-friendliness of youth clinics in northern Sweden: a survey analyzing the perspective of the youth

**DOI:** 10.1186/s12913-020-05188-4

**Published:** 2020-04-23

**Authors:** Anna-Karin Waenerlund, Miguel San Sebastian, Anna-Karin Hurtig, Maria Wiklund, Monica Christianson, Isabel Goicolea

**Affiliations:** 1 Department of Epidemiology and Global Health, Umeå University, 901 87 Umeå, Sweden; 2 Department of Community Medicine and Rehabilitation, Physiotherapy, Umeå University, Umeå, Sweden; 3 Department of Nursing, Umeå University, Umeå, Sweden

**Keywords:** Youth clinic, Sweden, Youth friendly, Health services, Health service accessibility, Youth, Questionnaire

## Abstract

**Background:**

Sweden has nearly 300 youth clinics that have been offering services since the 1970s. However, no evaluation has been done to assess their youth-friendliness. This study aims to assess: i) to what extent youth clinics are perceived as youth-friendly by the young people using them; and ii) if the level of youth friendliness is equally perceived across different sociodemographic groups of users.

**Methods:**

The four northernmost counties of Sweden were included in the study. Of the total identified 22 youth clinics, 20 participated by giving out questionnaires to the youth after their visits to the respective youth clinics. In total 1110 youth participated in the study and answered questions according to the World Health Organization’s criteria of accessibility, equity, respect, privacy and confidentiality, no judgement, and quality. Means and frequencies were calculated, and t-test and ANOVA were used to compare means by sociodemographic variables.

**Results:**

Participants perceived the youth clinics as very youth friendly across the measured domains, with scores as high as 4.8 and 4.9 (of a maximum of 5). Youth clinics were perceived in a similar way regardless of gender, but other sociodemographic factors influenced some of the domains, especially ethnic background.

**Conclusions:**

The perception of youth friendliness in youth clinics was very high. Nonetheless, younger users; users who did not categorize themselves as either heterosexual, homosexual, or bisexual; users with trans-experiences; and users with non-Swedish backgrounds gave youth clinics lower scores for certain domains.

## Background

Health care services can play an important role in the promotion of youth health; they can offer information and care to youth and promote healthy behaviour [1–3]. However, in order to play such a beneficial role, young people have to access such services and receive care that is judged to be of good quality and relevant for their needs; what the World Health Organization (WHO) conceptualizes as youth friendly health care services [3]. In order to be youth-friendly, services should fulfil the criteria of being accessible, acceptable, equitable, appropriate, and effective for different youth subpopulations [3, 4]. While WHO defines youth as those between 15 and 24 [5], the Youth Law (2004) of Sweden defines youth as those between 13 and 25 years.

While interventions to make services more youth-friendly have been implemented in many settings, few countries have truly integrated such an approach in a sustainable way within the health care system [2, 6, 7]. Head Space in Australia has implemented around 100 clinics, the Jigsaw clinics in Ireland account for 10 and Canada has around 12 of such services [8, 9], all of them with a special focus on integrating first line mental health care for young people. Sweden, on the other hand, has around 300 youth clinics (YCs) spread across the country and with more than 40 years of experience offering differentiated services for adolescents and youth [7, 10, 11]. While the target group of YCs is youth - according to the Swedish Youth Law definition- there are variations between YCs in terms of both lower and upper age limits [11]. According to the guidelines of The Swedish Society for Youth Centres (FSUM), and the perceptions of professionals working on YCs, they constitute an example of youth-friendly services [12]. Moreover their integration within the health system, while at the same time keeping a certain level of autonomy, makes them a good case for how youth-friendly services can be sustained within a broader health care system [11].

However, YCs are not without challenges. For example, it is well known that young women outnumber young men in YCs’ consultations, and professionals working in YCs point out that inequities might exist in terms of lack of access for certain groups of youth, such as LGBTQ and non-Swedish youths [11]. Also, the resources available differ geographically. YCs located in smaller places have fewer professionals and much shorter working hours, and many rural municipalities lack YCs [11, 13]. While YCs have always implemented a holistic youth-centred approach, the traditional focus has been on sexual and reproductive health. How to strengthen the role of YCs in other areas of health, especially mental health, is currently being discussed [13–15].

Despite the long history of YCs in Sweden, and even if there are internal reports stating that young people using the YCs are in general satisfied with the services provided [12], there is no published evaluation assessing to what extent YCs fulfil the domains of youth-friendliness, and whether these domains might vary for different youth subpopulations. Thus, this study had two objectives: i) to assess to what extent youth clinics were perceived as youth-friendly by the young people using them, and ii) if the level of youth-friendliness was equally perceived across different sociodemographic groups of users.

## Methods

### Study design and participants

This study was conducted in the four northernmost counties of Sweden, namely Jämtland-Härjedalen, Norrbotten, Västerbotten, and Västernorrland. Northern Sweden is a sparsely populated area; while accounting for 60% of Swedish land, it is home to only 12% of the population. Most people are clustered in the coastal regions whilst fewer people are situated inland. We focused on this region because it remains under-researched and its rurality better reflects the situation of other rural, scarcely populated areas in the European Union and beyond.

Of the 280 YCs in Sweden at the time of this study, 22 were located in northern Sweden and 20 participated in this study. Youth over the age of 16 who visited one of these 20 YCs were invited to fill in the YFHS-Swe questionnaire after the consultation. The questionnaire was completed in a quiet area at the YCs. The YFHS-Swe questionnaire assesses diverse domains of youth friendliness, based on the YFHS-WHO+ [4] and it has been validated for the Swedish context [16, 17].

In September 2016 the questionnaires were sent out to the YCs, and in March 2017 when the data collection ended, a total of 1110 young persons had responded. We excluded 113 responses due to the respondents’ being under age 16 or to their declining participation. Only five of the participating YCs kept track of how many youths declined to participate, and their response rate was between 70.3 and 90.9%. For the rest of the YCs, we could not state the response rate.

### Measures

#### Youth friendliness

The YFHS-Swe questionnaire assesses six main domains, namely: *accessibility, equity, respect, privacy and confidentiality, no judgement,* and quality, of which three have subdomains. The questionnaire can be found in Baroudi et al.’s [16].

Accessibility includes the subdomains of (a) contact (*access: contact*), (b) sexual and reproductive health (*access: sexual*), and (c) psychosocial health (*access: psychosocial*); equity has the subdomains of (a) diversity (*equity: diversity*) and (b) legal status (*equity: legal*); and quality has the subdomains of (a) quality of consultation (*quality: consultation*) and (b) quality of the facility (*quality: facility*).

All 10 domains and subdomains analysed in this article were assessed using Likert-type scales ranging from 1 = least youth friendly to 5 = most youth friendly - the specific items within each question were assessed from 1 to 5, being 1 = never present in the clinic to 5 = always present in the clinic. Table 1 shows a short description of the 10 subdomains contained in the YFHS-Swe questionnaire.

**Table 1:** Youth-friendliness subdomains assessed in the YFHS-Swe questionnaire (modified from [16])

Subdomain	Description
Access	
*Access sexual*	Ability to receive help related to sexual and reproductive health
*Access psychosocial*	Ability to receive help related to psychosocial health
*Access contact*	Ability to get contact and ease of accessing the service
Equity	
*Equity diversity*	Equal terms for youth disregards social or cultural background, gender, disability or other
*Equity legal*	Equal terms for youth with legal concerns
Privacy and confidentiality	The visit ensured confidentiality and privacy
No judgement	The staff provided attention, support and were non-judgemental
Respect	The youths felt that they are treated with respect
Quality	
*Quality consultation*	Quality of the encounter between staff and youth
*Quality facility*	Quality of the facility and information

#### Demographic factors

*Gender* was coded as women, men, and other (intergender, non-binary, and other). Trans-experience was dichotomized into yes and no. Sexual orientation included heterosexual, homosexual, bisexual, and other (queer, asexual, I don’t categorize myself sexually, I don’t know, and other). Place of birth was coded as being born in Sweden or outside the country and parents place of birth was classified as both parents born in Sweden, both born in Europe (but not in Sweden), or at least one of them born outside Europe.

### Analysis

To examine the participants’ perception of the YCs’ friendliness, different scores for each of the domains and the sum of all were created. For each domain, means were obtained by summing up the Likert responses and dividing the results by the number of items in each respective domain. The mean of all of the 10 factor scores was calculated and labelled “*Friendliness*.” Only full cases were analysed, and the response option “I don’t know” was excluded. To achieve the second objective, an analysis of variance was performed to assess whether the sociodemographic variables were associated with the mean of the 10 domains. A Bonferroni post-hoc test was also conducted to examine differences between the groups of variables. All analyses were performed in Stata 15.

## Results

Young people responding to the questionnaire were mostly young women (90.7%), heterosexual (84.4%), not reporting trans-experiences (98.60%), born in Sweden (93.99%), and with both parents born in Sweden (92.59%). Around one third of participants belonged to each of the different age groups (Table 2). Almost 15% were visiting the clinic for the first time.

**Table 2:** Characteristics of the participants

	N	%
Gender		
Women	985	90.7
Men	93	8.6
Other	8	0.7
**Age**		
16–17 years	348	32.7
18–19 years	325	29.3
20 and > years	390	35.1
**Sexuality**		
Heterosexual	937	84.4
Bisexual	82	7.4
Homosexual	9	0.8
Other	59	5.3
**Trans-person expression**		
No	1059	98.60
Yes	15	1.4
**Place of Birth**		
Sweden	1032	93.99
Outside Sweden	66	6.01
**Place of parents birth**		
Both in Sweden	925	92.59
Both in Europe	20	2.00
At least one outside Europe	54	5.41

**Fig. 1:**
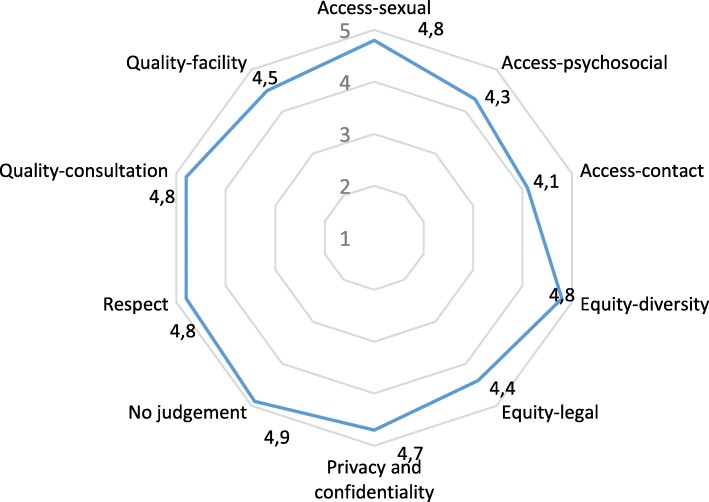
Mean scores ranging from 1 to 5 for the 10 subdomains of youth friendliness (effective *n* = 601–976)

Figure 1 shows that mean scores of youth-friendliness were overall very high. All domains rated above 4. *Access: contact* had the lowest score (4.1) and *non-judgment* had the highest (4.9).

Table 3 shows each domain’s mean scores according to the participants’ sociodemographic characteristics. No differences were found among most of the variables and the different domains. However, those above 19-years old were more satisfied with *access: contact*, *no judgment*, and *respect* than were the younger groups. Those categorized in the group ‘other’ regarding their sexual orientation gave lower scores in *access: sexual* and *respect* than those who identified themselves as heterosexual, homosexual, or bisexual. Those reporting trans-experiences reported a lower satisfaction with *access: sexual*, but higher with *access: contact*.

**Table 3:** Mean scores (and standard deviations, sd) in 10 subdomains of youth friendliness and its association with sociodemographic variables. Analysis of variance (ANOVA) was used to calculate the statistical inferences

	Access-Sexual	Access-Mental	Access- Contact	Equity- Diversity	Equity - Legal	Privacy	No judgement	Respect	Quality-Consultation	Quality Facility
**Gender**	Mean (sd)	Mean (sd)	Mean (sd)	Mean (sd)	Mean (sd)	Mean (sd)	Mean (sd)	Mean (sd)	Mean (sd)	Mean (sd)
Women	4.8 (0.5)	4.2 (0.8)	4.1 (0.8)	4.7 (0.5)	4.4 (0.9)	4.7 (0.6)	4.9 (0.4)	4.8 (0.5)	4.8 (0.5)	4.5 (0.7)
Men	4.7 (0.7)	4.3 (0.9)	4.3 (0.8)	4.6 (0.9)	4.4 (1.0)	4.7 (0.8)	4.8 (0.7)	4.7 (0.8)	4.7 (0.8)	4.6 (0.7)
Other	5.0 (0.1)	4.2 (1.1)	4.2 (0.8)	4.9 (0.4)	4.8 (0.4)	4.6 (0.6)	4.9 (0.1)	4.8 (0.3)	5.0 (0.0)	4.4 (1.1)
**Age**										
16–17 years	4.8 (0.6)	4.2 (0.9)	4.0 (0.8)	4.8 (0.6)	4.3 (0.9)	4.6 (0.6)	4.8 (0.4)	4.7 (0.5)	4.8 (0.5)	4.5 (0.7)
18–19 years	4.8 (0.5)	4.2 (0.9)	4.0 (0.8)	4.7 (0.6)	4.4 (0.9)	4.6 (0.6)	4.8 (0.5)	4.7 (0.5)	4.8 (0.6)	4.5 (0.7)
20 and > years	4.9 (0.4)	4.3 (0.8)	4.3 (0.7)*	4.7 (0.6)	4.4 (0.9)	4.7 (0.5)	4.9 (0.4)*	4.8 (0.4)*	4.8 (0.4)	4.4 (0.7)
**Sexuality**										
Heterosexual	4.8 (0.5)	4.2 (0.9)	4.1 (0.8)	4.7 (0.6)	4.4 (0.9)	4.7 (0.6)	4.9 (0.4)	4.8 (0.5)	4.8 (0.5)	4.5 (0.7)
Homosexual	5.0 (0.0)	4.3 (0.8)	4.1 (0.7)	4.7 (0.7)	4.9 (0.2)	5.0 (0.0)	5.0 (0.0)	5.0 (0.0)	5.0 (0.0)	4.7 (0.5)
Bisexual	4.8 (0.5)	4.8 (0.4)	4.2 (0.9)	4.9 (0.0)	4.4 (1.0)	4.6 (0.7)	4.8 (0.6)	4.7 (0.6)	4.7 (0.7)	4.4 (0.8)
Other	4.7 (0.7)*	4.1 (0.8)	4.3 (0.8)	4.6 (0.8)	4.3 (1.0)	4.5 (0.8)	4.7 (0.6)	4.6 (0.7)*	4.7 (0.7)	4.6 (0.5)
**Trans person experiences**										
No	4.8 (1.1)	4.2 (0.9)	4.1 (0.8)	4.7 (0.6)	4.4 (0.9)	4.7 (0.6)	4.9 (0.4)	4.8 (0.5)	4.8 (0.5)	4.6 (0.7)
Yes	4.4 (0.5)*	3.9 (0.9)	4.5 (0.7)*	4.5 (1.3)	4.2 (1.4)	4.7 (0.5)	4.9 (0.2)	4.9 (0.3)	4.9 (0.3)	4.5 (0.9)
**Birth place**										
Sweden	4.8 (0.5)	4.2 (0.8)	4.1 (0.8)	4.7 (0.6)	4.4 (0.9)	4.7 (0.6)	4.9 (0.4)	4.8 (0.5)	4.8 (0.5)	4.5 (0.7)
Outside Sweden	4.7 (0.6)*	4.1 (0.8)	4.2 (0.7)	4.5 (0.9)*	4.3 (0.9)	4.5 (0.8)*	4.8 (0.7)	4.7 (0.7)	4.7 (0.7)	4.4 (0.8)
**Parents birth place**										
Both in Sweden	4.8 (0.5)	4.2 (0.8)	4.1 (0.8)	4.7 (0.6)	4.4 (0.9)	4.7 (0.6)	4.9 (0.4)	4.8 (0.4)*	4.8 (0.5)	4.8 (0.5)
Both in Europe	4.9 (0.2)	4.2 (0.5)	4.1 (0.8)	4.7 (0.5)	4.1 (0.9)	4.6 (0.4)	4.9 (0.2)	4.8 (0.8)	4.8 (0.4)	4.8 (0.4)
At least one outside Europe	4.6 (0.7)	4.0 (0.9)	4.0 (0.9)	4.4 (1.0)*	4.3 (1.1)	4.3 (1.2)*	4.5 (1.1)*	4.4 (1.1)*	4.5 (1.1)*	4.5 (1.1)*

* *P*-value ≤0.05

Youth born outside Sweden reported less satisfaction with *access: sexual*, *equity: diversity*, and *privacy and confidentiality*. In addition to the last two, *no judgment*, *respect*, *quality: consultation* and *quality: facility* scored lower when at least one of the parents was born outside Europe.

## Discussion

To the extent of our knowledge this is the first study to assess Swedish YCs’ degree of youth-friendliness from the perspective of young people using these services. The participants perceived the YCs as very youth friendly across the measured domains. YCs were perceived in a similar way regardless of the respondents’ gender, but other sociodemographic factors influenced some of the domains.

The YCs were overall assessed very positively by the young people answering the questionnaire. This is an important finding that allows us to label northern Swedish YCs as a good example of youth-friendly services. There are, to the best of our knowledge, no similar studies in other countries to compare with so far. However, during the validation of the YFHS+ questionnaire with primary health care centres in Bosnia-Herzegovina, the scores were considerably lower there [4]. Results from our study confirm previous unpublished evaluations from FSUM and findings from qualitative studies that stress that the “special” youth-centred approach of YCs and the motivation of the staff working on these services make them accessible, acceptable, and appropriate for young people [11, 12]. Since there are few examples of existing and sustained youth-friendly services in Europe [18, 19], the lessons learnt from the Swedish YC model can inspire efforts in other countries.

In terms of differences based on the sociodemographic characteristics of youths, it is interesting to highlight that there were no significant differences based on gender. Studies, and routine data from YCs’ consultations (and even the composition of our sample) highlight that girls and young women outnumber boys and young men in consultations in YCs [20]. This is a pattern not only for Swedish YCs, but for youth-friendly health care services in general. This study, however, also points out an interesting finding: when it comes to those youths actually attending YCs, boys and young men (and also those who do not categorize themselves in gender binary ways) perceive all domains of YCs as high as do girls and young women.

In relation to girls and young women it is also important to note that they rate YCs high in the different domains of youth friendliness. It is known that teenage girls and young women take high responsibility for sexual health and contraceptives in partner relationships [21]. In addition, young women are overrepresented in sick-leave and self-reported health problems (e.g. mental health problems), as well as in exposure to gender-based and sexualised harassment and violence [22, 23], which is why youth-friendly strategies for continuous, early health promotion are important to develop for these groups.

Sexual orientation was one aspect that influenced how young people perceived YCs. The literature shows that LGBTQ youth face barriers to accessing health care services [24–27]. However, most studies take together as a group all non-heterosexual youths. In our study, there were no significant differences between heterosexual, homosexual and bisexual youths, while queer, asexual and non-sexual youth as well as youth with trans experiences rated YCs’ differently—and generally lower. This could reflect the fact that while training, LGBTQ certifications, and other efforts might have had an impact in how health care services for youth address sexual diversity, youth with less normative sexualities (beyond the heterosexual, homosexual, bisexual categorizations) still face increased barriers for accessing services.

The young people’s, and especially their parents’, country of birth were the variables that were most strongly associated with YCs’ lower rating in the different domains. The literature shows that migrants might face more barriers to accessing health care services, based on individual characteristics -e.g. socioeconimc status, language and information barriers -and, especially based on factors at the health-system level -e.g, policies that restrict access, and health care professionals’ attitudes, such as discrimination and racism [28–31]. Despite equity being in the core of the YCs’ mandate, previous studies have highlighted that YCs’ staff perceive that non-Swedish young people access YCs to a lesser extent [11]. This study goes further, pointing out that for those young people with a non-Swedish background who actually reach YCs, their perceptions of the services are also a bit poorer.

Equity is a domain of youth-friendliness that other studies show as being among the most difficult to fulfil [2, 6, 11]. While previous population-based studies have already pointed out that there are socioeconomic inequities in accessing YCs in Sweden [32], and YC staff’s perceptions support the hypothesis that certain sub-groups of young people access YCs much less [11, 12, 32], this study reveals that sexual orientation and especially ethnic background are markers of inequities when it comes to YCs’ youth-friendliness.

According to the new proposed model by WHO, it is not only a matter that specific, differentiated services should be friendly towards young people, but that the entire health system should embrace such an approach [33]. It would be interesting to apply the YFHS-Swe questionnaire to other health care services that also assist young people in Sweden (primary health care services, youth psychiatry, school health) in order to assess whether they are equally youth friendly.

Finally, it is important to highlight that the sample does not represent the overall young population in Sweden (women, heterosexual, born in Sweden youth are overrepresented). We hypothesize that this, more than being a bias in the selection of the participants, is a reflection of who access youth clinics, and who does not. Special attention should therefore be given to implement strategies to improve access to certain subgroups of young people who might be in more need but accessing clinics less. There are a number of strategies in place when it comes to improving access to certain subgroups of youth, i.e. LGBTQ certification of clinics, visits of students when they are in 9th grade (15–16 years), tailored information and specific drop-in hours addressing those identifying themselves as boys/young men, and certain clinics collaborate with organizations working with unaccompanied youth. However, we claim that more measures need to be taken, including better promotion of the existence and services provided in YCs for all young people, especially for rural youth and those who have moved to Sweden recently and might not be familiar with the services. Crucial is also to remove any barriers related to payment (i.e. for young people who have not been granted legal residence). To date, there are some promising initiatives to overcome barriers and improve access such as the possibility to receive support via digital technologies (i.e. psychologist), including a web-based youth health clinic which is partly translated to additional languages (www.youmo.se).

### Methodological considerations

The distribution of the questionnaires within the YCs might have differed and youth who were perceived to be less satisfied or who were not fluent in Swedish might have been excluded. Internal missing was evident in most questions. This study was only able to capture the visiting youths’ perceptions, and not other youths’ perceptions. Moreover, we were not able to gather information on how many young people declined to answer the survey and their sociodemographic characteristics.

## Conclusions

For all the youths participating in the study, the perception of youth friendliness in YCs was very high, scoring almost the maximum for *access: sexual*, *equity: diversity*, *privacy and confidentiality*, *no judgement*, *respect*, and *quality: consultation*. YCs received lower scores in certain domains from younger users; from those who did not categorize themselves as either heterosexual, homosexual, or bisexual; from those with trans-experiences; and from those with non-Swedish backgrounds.

The use of the WHO criteria and the YFHS-Swe questionnaire is a good, promising way to scrutinize additional services that meet youth in their daily practices (such as primary care, psychiatry, school health services, paediatric clinics, pain rehabilitation clinics, stress clinic, dental care, etc.).

## Data Availability

The datasets used and/or analysed during the current study are available from the corresponding author on reasonable request.

## Abbreviations

FSUM: Föreningen för Sveriges Ungdomsmottagningar (Swedish Association of Youth Clinics)
LGBTQ: Lesbian Gay Bisexual Trans Queer
WHO: World Health Organization
YC: Youth Clinic
YFHS-Swe: Youth Friendly Health Services Sweden
YFHS+: Youth Friendly Health Services Plus
